# The impact of digital transformation on green total factor productivity of heavily polluting enterprises

**DOI:** 10.3389/fpsyg.2023.1265391

**Published:** 2023-11-03

**Authors:** Jiabin Han, Ruyu Sun, Muhammad Zeeshan, Alam Rehman, Irfan Ullah

**Affiliations:** ^1^School of Business Administration, Liaoning Technical University, Huludao, China; ^2^Faculty of Management Sciences, National University of Modern Language, Islamabad, Pakistan; ^3^Reading Academy, Nanjing University of Information Science and Technology, Nanjing, China

**Keywords:** digital transformation, green total factor productivity, green innovation, management efficiency, external transaction costs

## Abstract

**Introduction:**

Digital transformation has become an important engine for economic high-quality development and environment high-level protection. However, green total factor productivity (GTFP), as an indicator that comprehensively reflects economic and environmental benefits, there is a lack of studies that analyze the effect of digital transformation on heavily polluting enterprises’ GTFP from a micro perspective, and its impact mechanism is still unclear. Therefore, we aim to study the impact of digital transformation on heavily polluting enterprises’ GTFP and its mechanism, and explore the heterogeneity of its impact.

**Methods:**

We use Chinese A-share listed enterprises in the heavily polluting industry data from 2007 to 2019, measure enterprise digital transformation indicator using text analysis, and measure enterprise GTFP indicator using the GML index based on SBM directional distance function, to investigate the impact of digital transformation on heavily polluting enterprises’ GTFP.

**Results:**

Digital transformation can significantly enhance heavily polluting enterprises’ GTFP, and this finding still holds after considering the endogenous problem and conducting robustness tests. Digital transformation can enhance heavily polluting enterprises’ GTFP by promoting green innovation, improving management efficiency, and reducing external transaction costs. The improvement role of digital transformation on heavily polluting enterprises’ GTFP is more obvious in the samples of non-state-owned enterprises, non-high-tech industries, and the eastern region. Compared with blockchain technology, artificial intelligence technology, cloud computing technology, big data technology, and digital technology application can significantly improve heavily polluting enterprises’ GTFP.

**Discussion:**

Our paper breaks through the limitations of existing research, which not only theoretically enriches the literature related to digital transformation and GTFP, but also practically provides policy implications for continuously promoting heavily polluting enterprises’ digital transformation and facilitating their high-quality development.

## 1. Introduction

The Outline of the 14th Five-Year Plan and the Long-Range Objectives Through the Year 2035 points out that we should “promote the green transformation of key industries and important fields.” In accordance with the Second National Pollution Source Census Bulletin, only five heavily polluting industries, such as the metal products industry, constitute up to 44.14% of China’s total industrial pollution sources. As we can see, being the major players in energy consumption and environmental pollution, improving the GTFP of heavily polluting enterprises has become an important part of achieving the strategic objective of “double carbon” and high-quality economic development (Wang et al., 2022; Li K. et al., 2023). However, heavily polluting enterprises face many problems, such as low resource utilization rate (Lu and Li, 2023), insufficient motivation for green innovation (Xie et al., 2022), high environmental protection pressure, and high regulatory costs (Long et al., 2022; Liu S. et al., 2023), which severely restrict their GTFP improvement. Therefore, how to effectively improve heavily polluting enterprises’ GTFP has become a problem that needs to be addressed at present.

Digital transformation is becoming a leading force in empowering traditional industries’ green upgrades (Tian et al., 2022; Zhang W. et al., 2023). The 14th Five-Year Plan for the Development of the Digital Economy points out that we should “vigorously promote industry digital transformation” and “promote green development in digital transformation process.” In accordance with the prediction of the World Economic Forum, the carbon emissions reduced by industries benefiting from digital technology will be as high as 12.1 billion tons by 2030. In order to seize digital development opportunities, many enterprises are rapidly promoting digital transformation with advanced digital technologies such as artificial intelligence (AI), blockchain (BD), cloud computing (CC), and big data (DT) as the core (Wu et al., 2021; Shang et al., 2023). According to the China Enterprise Digital Transformation Research Report (2022), more than 81.0% of surveyed enterprises are in the full optimization stage of digital transformation, meanwhile, the number of enterprises in the initial construction stage of digital transformation grows to 16.9%. Heavily polluting enterprises use advanced digital technologies to transform their own business in an all-round, multi-angle, and full-chain transformation, and improve the digital level of manufacturing, R&D innovation, operation management, and other links, which is conducive to optimizing production methods, improving innovation ability and improving energy consumption structure (Li and Wang, 2022; Sheng et al., 2022; Li G. et al., 2023; Tian et al., 2023), thus providing strong kinetic energy for improving GTFP. Therefore, this paper focuses on whether digital transformation can improve heavily polluting enterprises’ GTFP. What are the underlying mechanisms? What is the heterogeneity of its impact? The answers to these questions are of great significance in accelerating enterprise digital transformation and promoting its green transformation.

Although existing research has paid much attention to GTFP influencing factors and digital transformation microeconomic effects, there are still gaps that need to be improved. First, the relationship between digital transformation and heavily polluting enterprises’ GTFP is not yet clear. Existing studies have focused on exploring the influence of digital economy and digital finance on GTFP from the regional and industry perspectives (Sun et al., 2022; Chen et al., 2023; Han et al., 2023; Hao et al., 2023; Lee et al., 2023; Liu D. et al., 2023), and some studies have also explored the influence of digital transformation on enterprise total factor productivity (TFP), pollution reduction, and environmental performance from the enterprise level (Du and Jiang, 2022; Ren et al., 2022; Cheng et al., 2023; Liu M. et al., 2023; Su et al., 2023; Xu et al., 2023). However, enterprise GTFP, as a comprehensive indicator of its productivity and environmental performance, can more accurately reflect its high-quality development, there are almost no studies examining its impact on heavily polluting enterprises’ GTFP based on enterprise digital transformation perspective. Second, the mechanism by which digital transformation affects heavily polluting enterprises’ GTFP needs to be explored. Currently, the theoretical system of digital transformation and enterprise GTFP is not sound, and the channels through which digital transformation influences heavily polluting enterprises’ GTFP is a worthy work to be further explored. Third, the asymmetric effect of digital transformation on heavily polluting enterprises’ GTFP needs to be discussed. There is no study that examines how digital transformation impacts heavily polluting enterprises’ GTFP based on enterprise property rights, industry science and technology attributes, geographic regions, and structural characteristics. Therefore, Chinese A-share listed companies in the heavily polluting industry from 2007 to 2019 are taken as the research sample to investigate the effect of digital transformation on heavily polluting enterprises’ GTFP and its mechanism, as well as to explore the characteristics of heterogeneity exhibited by its impact. In comparison with previous research, the possible marginal contributions of this study include three aspects: First, digital transformation and GTFP are brought into a unified research framework from the perspective of micro-enterprises, and analyze the effect of digital transformation on heavily polluting enterprises’ GTFP, which expands the research of microeconomic effects of digital transformation as well as provides a new way for improving heavily polluting enterprises’ GTFP. Second, the mechanism of digital transformation affecting heavily polluting enterprises’ GTFP is clarified from the channels of green innovation, management efficiency, and external transaction costs, which opens the “black box” of the mechanism between them as well as provides empirical evidence for enhancing heavily polluting enterprises’ GTFP through digital transformation. Third, the heterogeneous impact of digital transformation on heavily polluting enterprises’ GTFP is explored from the perspectives of enterprise property rights nature, industry science and technology attributes, geographic regions, and structural characteristics of digital transformation, which enriches the relevant studies and provides important references for the government in formulating refined digital policies.

The subsequent organization of this paper is as follows. Section 2 reviews the relevant literature. Section 3 conducts the theoretical analysis. Section 4 introduces the research methodology. Section 5 provides the empirical analysis. Section 6 summarizes the conclusions and makes suggestions.

## 2. Literature review

Unlike traditional TFP, which focuses only on the contribution of capital and labor inputs to desired outputs such as economic efficiency, GTFP also includes energy consumption and environmental pollution as inputs and non-desired outputs, respectively, into the accounting system (Li et al., 2013; Lee and He, 2022), which can accurately reflect the high-quality development. Research on GTFP mainly focuses on its measurement methods and influencing factors. With respect to the measurement methods, Chung et al. (1997) firstly measured GTFP including non-desired outputs such as pollutants by using the directional distance function (DDF) and decomposed the efficiency by using the ML index method, and Tone (2001) further improved the method by establishing the directional distance function on the basis of the slack variables (SBM-DDF). Later, Fukuyama and Weber (2009) combined the SBM-DDF with the ML index method to measure GTFP. However, the ML index does not have the transferability and circularity, and may be unsolved across periods. Oh (2010) proposed the GML index, which can make up for the shortcomings of the ML index. With respect to the influencing factors, some studies have explored the influencing factors of GTFP from both the enterprise’s internal and external levels. With regard to the influencing factors, established studies have explored enterprise GTFP influencing factors at both enterprise internal and external levels. From the view of internal factors, enterprise green innovation (Wu J. et al., 2022) has a significantly enhancing impact on enterprise GTFP. From the view of external factors, positive investor sentiment (Zhao and Yan, 2023), urban environmental legislation (Zhang et al., 2022), green credit policy (Lv et al., 2023), and regional social capital (Sun et al., 2022) can effectively improve enterprise GTFP, while regarding the effects of environmental regulations, existing studies come to three different types of conclusions, one is that environmental protection tax can improve enterprise GTFP (Tian and Feng, 2022), the other is that carbon emission permit trade mechanism will inhibit the improvement of enterprise GTFP (Hu and Ding, 2020), and the third is that the impact of environmental regulations on enterprise GTFP is non-linear (Ju et al., 2020). As for the relationship between digitization and GTFP, the existing literature mainly analyzes the role of digitization on GTFP at the regional and industry levels. Most scholars believe that digitization has an obvious enhancement effect on GTFP, digitization helps to enhance China’s Yangtze River Delta cities’ GTFP (Lee et al., 2023). The digital economy not only directly increases GTFP in local areas, but also has spillover effects on neighboring areas (Sun et al., 2023). Digital financial inclusion is able to increase GTFP in rural areas (Liu D. et al., 2023), and digital finance can offset some of the negative effects of environmental regulations, and overall show the effect of raising GTFP (Han et al., 2023). From an industry perspective, the digital economy can realize an increase in manufacturing GTFP by raising technological efficiency (Hao et al., 2023). In addition, Chen et al. (2023) found that the digital economy has an inverted U-shaped effect on regional GTFP. However, few studies have examined its relationship with heavily polluting enterprises’ GTFP from the microenterprise digital transformation perspective.

Digital transformation refers to the systematic process of data-driven, deep integration of advanced digital technologies with core links of enterprises to promote changes in production methods, business processes, and business model reorganization, ultimately achieving improved enterprise efficiency and empowering enterprise transformation and upgrading (Warner and Wäger, 2019; Gilch and Sieweke, 2021). Academics have explored the measurement methods and microeconomic effects of digital transformation, and have drawn rich conclusions. With regard to measurement methods, there are currently three mainstream methods: first, the dummy variable method (Peng and Tao, 2022), in which the enterprise carries out digital transformation as 1 and the opposite 0, but the method only reflects whether or not the enterprise carries out digital transformation, and cannot reflect digital transformation degree; second, the single indicator method (Cheng et al., 2023), which is measured by the ratio of the total amount of ICT hardware and software capital to the total assets, but the method only focuses on particular capital applications, and in fact, digital transformation also requires other capital inputs, so its measurement results may be underestimated; third, the text analysis method (Wu et al., 2021; Yuan et al., 2021; Du et al., 2023), which is measured by analyzing digitization-related words frequency in companies’ annual reports, and it can reflect digital transformation actual situation in a more comprehensive way than the two methods mentioned above. With regard to microeconomic effects, most scholars focus on the influence that digital transformation brings to enterprises themselves. First of all, digital transformation can bring efficiency changes to enterprises, which can enhance their innovation ability and crack the innovation dilemma (Liu M. et al., 2023; Zhuo and Chen, 2023), help improve their performance (Peng and Tao, 2022; Zhai et al., 2022; Guo et al., 2023; Zhang Y. et al., 2023), facilitate specialized division of labor (Yuan et al., 2021), and exert good governance effectiveness to contribute to improving corporate governance (Qi et al., 2020). Meanwhile, digital transformation also has a clear influence on enterprise TFP. The general view is that digital transformation can enhance enterprise TFP by reducing costs, promoting innovation, improving operations, and optimizing human capital (Du and Jiang, 2022; Ren et al., 2022; Su et al., 2023), additionally, Cheng et al. (2023) found that the relationship between the two is U-shaped. Secondly, digital transformation can also bring green changes to enterprises, which can raise their green innovation level (Feng et al., 2022; He et al., 2023; Xiao and Zeng, 2023), contribute to carbon reduction (Shang et al., 2023), reduce overall pollution emissions (Li G. et al., 2023), and ultimately improve environmental performance (Xu et al., 2023). In addition, some scholars have also found that digital transformation has significant positive effects on external capital markets, which can effectively enhance stock liquidity levels (Wu et al., 2021) and reduce the stock price crash risk (Wu K. et al., 2022). However, established research ignores the influence of digital transformation on heavily polluting enterprises’ GTFP.

In summary, existing studies have richly explored around GTFP and digital transformation, providing method reference and theoretical support for our study. First, in terms of research methods, the GML index based on the SBM directional distance function is proved to be able to avoid the result bias caused by the radial and angular problems, and also realize the global comparability of the production frontiers, so we adopt this method to measure GTFP. Meanwhile, due to the simplicity and accuracy of the text analysis method, which is able to more comprehensively reflect the digital transformation situation, we adopt this method to measure digital transformation. Second, in terms of research perspectives, existing studies have confirmed that digitalization has a positive role on GTFP at the regional and industry levels, and also affirmed the positive role of digital transformation on efficiency and green development at the enterprise level, but few studies have analyzed digital transformation and GTFP in a unified framework from the enterprise level, which provides a research direction and space for our paper, therefore, we focus on the enterprise perspective to consider the influence of digital transformation on heavily polluting enterprises’ GTFP. Third, in terms of mechanism, previous studies have proved that digital transformation has positive effects on enterprise green innovation (Feng et al., 2022; He et al., 2023; Xiao and Zeng, 2023), governance level (Qi et al., 2020), and specialized division of labor (Yuan et al., 2021), which helps to lay the foundation for the theoretical analysis of our paper, so we focus on the mechanism of green innovation, management efficiency, and external transaction costs.

## 3. Theoretical analysis

### 3.1. Digital transformation and GTFP

The essence of improving heavily polluting enterprises’ GTFP lies in maximizing business performance and minimizing environmental pollution with minimum inputs of capital, labor, and energy. Digital transformation is essentially the advancement of production technology and optimization of factor allocation in heavily polluting enterprises (Peng and Tao, 2022), which can promote their energy conservation and pollution reduction, cost reduction, and efficiency improvement, achieve green and efficiency changes, and fundamentally improve GTFP. First of all, the technological progress brought about by digital transformation can not only increase enterprise resource usage efficiency and reduce the waste of resources in production but also help to enhance production technology cleanliness and accelerate updating of production equipment, promoting enterprises to invest in even modern and environmentally friendly production machines, thus realizing energy conservation and pollution reduction (May et al., 2017). Second, enterprises embed digital technology and data elements into core business processes through digital transformation to empower management scenarios such as human resources, R&D innovation, capital operation, and supply chain, which is helpful to improve factor resource mismatch, making the enterprise’s production costs lower and enhancing the production efficiency (Liu S. et al., 2021). Among them, enterprises can transform and upgrade their business processes by means of advanced digital technology, which can help them transform from the traditional production system to the digital production system, reduce their costs and improve their efficiency (Wu et al., 2021). At the same time, enterprise digital transformation can enable more efficient use of data as a critical element, which can greatly enhance enterprise resource allocation efficiency and then enhance their production efficiency. On this basis, the hypothesis below is formulated:

*H1*. Digital transformation can effectively enhance heavily polluting enterprises’ GTFP.

### 3.2. Mechanisms of digital transformation affecting GTFP

#### 3.2.1. Green innovation mechanism

Digital transformation is helpful to promote green innovation and thus enhance heavily polluting enterprises’ GTFP. Emerging digital technology can effectively configure green innovation resources and optimize green innovation mode so as to promote heavily polluting enterprises’ green innovation. First of all, the application of emerging digital technologies makes it possible to broaden the access channels to green innovation resources and make the optimization of green innovation resource allocation (Ning et al., 2022) so as to enhance enterprise green innovation ability. Second, digital transformation can promote collaboration and knowledge sharing between enterprises and innovation subjects such as universities and research institutions and promote enterprises to shift from independent innovation mode to collaborative innovation mode, thus improving the efficiency of enterprise green innovation. According to green innovation, heavily polluting enterprises can achieve energy conservation and pollution reduction through two main ways: source control technology innovation and end treatment technology innovation, thus improving GTFP. From source control technology innovation, enterprises can use clean production technology at the source to save energy and curb pollutant generation; from end treatment technology innovation, enterprises can use waste treatment and energy-saving technology transformation at the end to increase the efficiency of energy usage and decrease the emission of pollutants (Xie and Han, 2022). Moreover, as enterprises improve their green innovation level, their production costs can be reduced significantly, and their production efficiency can be improved significantly, which ultimately helps to enhance GTFP. On this basis, the hypothesis below is formulated:

*H2*. Digital transformation can enhance heavily polluting enterprises’ GTFP by promoting green innovation.

#### 3.2.2. Management efficiency mechanism

Digital transformation is helpful to improve management efficiency and thus enhance heavily polluting enterprises’ GTFP. Advanced digital technology can optimize enterprise operation and management modes, improve communication and supervision efficiency, and thus improve management efficiency. Compared with the traditional mode, digital transformation is more focused on the penetration and integration of data resources, digital platforms, and digital technology with the field of enterprise management, which can help enterprises break the original management mode and transform from the traditional industrialized management mode to the digital management mode (Liu S. et al., 2021), directly enhancing enterprise management efficiency. Moreover, the introduction of an efficient communication management and information processing system through digital transformation is able to increase the coordination between internal departments, decrease the coordination costs between internal departments (Liu et al., 2020), and improve the communication efficiency between internal personnel when enterprises operate and manage and process information. Meanwhile, applying advanced digital technology in enterprises is able to increase the transparency and real-time monitoring of management processes such as finance and internal control, reduce the cost of supervision and efficiency loss due to the principal-agent problem (Chen and Kamal, 2016), and thus enhance enterprise management efficiency. Improving management efficiency is a significant way for heavily polluting enterprises to enhance GTFP. On the one hand, enterprises with high management efficiency can make optimal factor input decisions according to their operating conditions and changes in the external environment (Lev and Radhakrishnan, 2005), which is helpful to improve their own resource allocation and combination capabilities, thus improving their utilization efficiency of existing resources. On the other hand, enterprises with higher management efficiency are more capable of integrating internal innovation resources, which can optimize their innovation system as well as enhance their innovation level, thus improving GTFP. On this basis, the hypothesis below is formulated:

*H3*. Digital transformation can enhance heavily polluting enterprises’ GTFP by improving management efficiency.

#### 3.2.3. External transaction cost mechanism

Digital transformation is helpful to reduce external transaction costs and thus enhance heavily polluting enterprises’ GTFP. Digital transformation mainly reduces the heavily polluting enterprise external transaction costs from four points: First, digital transformation broadens the access to information and accelerates the speed of enterprise information processing, which enables enterprises to learn more quickly about the qualifications and products of counterparties and reach more upstream and downstream related enterprises (Malone et al., 1987), thus improving the market environment of incomplete and asymmetric information and decreasing the information enterprise search costs. Second, applying digital technology makes it possible for enterprises to access more transparent information about product prices, quality, and other critical elements of the contract, and increasing information transparency is able to help increase enterprise communication efficiency, which in turn reduces the negotiation costs between enterprises (Shi and Li, 2020). Third, enterprises can use digital technologies, including the Internet and the Internet of Things, to contact in time and track transaction status in real-time, and intervene in unintended situations caused by incomplete contracts and transactions deviating from the direction of cooperation, thus reducing the supervision costs in the process of contract signing and performance (Clemons et al., 1993). Fourth, the recording, storage, and dissemination of information brought by digital technology can help enterprises to match high-quality counterparties, effectively reduce the probability of counterparty default, and thus reduce the default costs of enterprises (Yuan et al., 2021). The reduction of external transaction costs is able to enhance enterprise GTFP in a significant way. Reducing external transaction costs can improve enterprises’ operational efficiency and profit margins and help promote resource allocation optimization, shifting from traditional manufacturing with low added value and high environmental pollution to service-oriented manufacturing with high added value and environmental friendliness. In addition, the reduction of external transaction costs can not only provide more adequate financial support for companies to undertake green technological innovation so as to alleviate their green transformation financing constraints but also help create a more steady and controllable external environment for them, which promotes them to focus efforts and resources on building their core competitive advantages (Du and Lou, 2022), creating favorable conditions for them to undertake green innovation activities so as to enhance GTFP. On this basis, the hypothesis below is formulated:

*H4*. Digital transformation can enhance heavily polluting enterprises’ GTFP by reducing external transaction costs.

**Figure 1:**
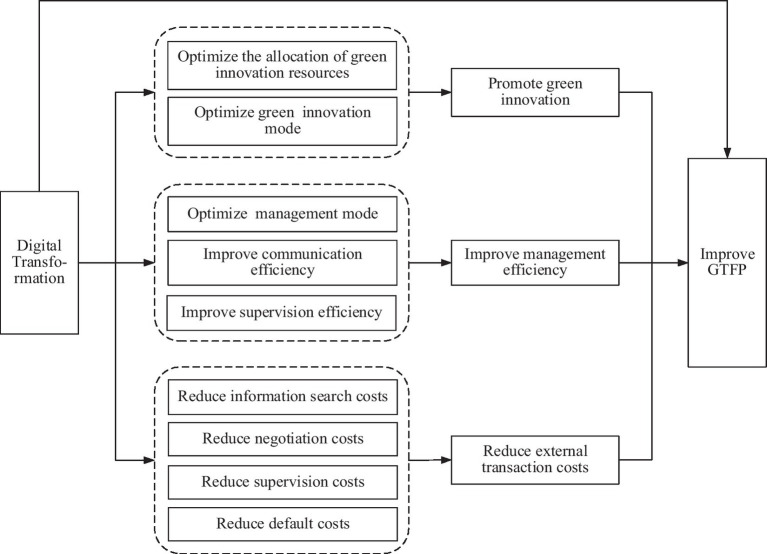
Theoretical analysis framework.

In summary, the overall theoretical framework of Figure 1 is constructed.

## 4. Methodology

### 4.1. Sample and data

Chinese A-share listed enterprises in the heavily polluting industry from 2007 to 2019 are selected as the sample in order to investigate the effect of digital transformation on heavily polluting enterprises’ GTFP. Drawing on Li and Xiao (2020) and Zhou et al. (2021) to select heavily polluting industries, 15 industries, including coal mining and washing industry, are classified as heavily polluting industries and screened in accordance with the following conditions: (1) Excluding ST and ST* firm samples; (2) Excluding the samples of missing core variables. Finally, we have acquired 3,107 valid observations. The industry codes include: B06, B09, C17, C18, C19, C22, C25, C26, C27, C28, C29, C30, C31, C32, D44. The data are obtained from annual reports of listed companies, CSMAR database, CNRDS database, China Energy Statistical Yearbook, China Statistical Yearbook, and China Environmental Statistical Yearbook. Additionally, to eliminate the outlier effect, our research applies 1% winsorizes to the major consecutive variables.

### 4.2. Variables

#### 4.2.1. Explained variable

Green total factor productivity (GTFP). Drawing on Oh (2010) and Liu J. et al. (2021), adopting the GML index based on the SBM directional distance function to represent enterprise GTFP. The indexes that measure enterprise GTFP include input and output indicators, and capital, labor, and energy are input indexes. The capital input adopts the enterprise capital stock which is measured by the perpetual inventory method to measure, that is, $K_{t}=K_{t-1}\left(1-\delta \right)+I_{t}/P_{t}$ , in which, $K_{t}$ , $K_{t-1}$ represent enterprise capital stock in period t and period t−1 , δ is the depreciation rate which takes 5%, It is the fixed asset investment indicator of period t , and $P_{t}$ is the fixed asset investment price index of period t in the province in which companies are established; labor input adopts the total number of staffs at year-end of the company to measure; energy input adopts enterprise energy consumption to express, and since enterprise energy consumption data can hardly be obtained directly, and considering that enterprise energy consumption has distinctive industry commonality, therefore, we calculate enterprise energy consumption in accordance with the industry energy consumption data, that is, $E_{k}=\sum {E_{k}}^{*}C_{k}/\sum C_{k}$ , where, $E_{k}$ denotes the energy consumption of enterprise K , $\sum E_{k}$ denotes industry energy consumption where enterprise K is established, $C_{k}$ denotes the operating cost of enterprise K , and $\sum C_{k}$ denotes the industry cost where enterprise K is established. Desired output and non-desired output are included in the output index. Enterprise operating income is adopted to represent desired output and deflated using their province’s industry producer ex-factory price index; non-desired output is expressed in terms of emissions of industrial sulfur dioxide, emissions of chemical oxygen demand from industrial wastewater, and production of industrial solid waste, for which the calculation method is the same as for energy inputs.

#### 4.2.2. Explanatory variable

Digital transformation (DCG). With reference to Wu et al. (2021), this research adopts text analysis to evaluate enterprise digital transformation. In particular, the text content in the annual reports of sample enterprises is extracted through the Python crawler function so as to build a data pool, and enterprise digital transformation characteristic word atlas is constructed from two aspects: “underlying technology” and “practical application,” in which the “underlying technology” level is divided into four subdivisions of AI, BD, CC and DT with “ABCD” technology as the boundary. The “practical application” level is digital technology application (ADT). On this basis, the word frequencies of the above-mentioned characteristic words of the data pool are counted by using the Chinese word segmentation function of Jieba, and the summed word frequency is logarithmized as an indicator to measure enterprise digital transformation.

#### 4.2.3. Control variables

Drawing on previous researches, our study selects control variables as follows: enterprise size (Size), enterprise age (Age), asset-liability ratio (Lev), profitability (ROA), capital expenditure (Capital), operating cost ratio (Cost), shareholding concentration (Share), and nature of ownership (SOE). The relevant variables are defined in detail in Table 1.

**Table 1:** Variable definitions.

Variable name	Variable symbol	Variable definition
Green total factor productivity	GTFP	Enterprise green total factor productivity measured by GML index on the basis of the SBM directional distance function
Digital transformation	DCG	Word frequency of words involving digital transformation feature words in corporate annual reports plus one to take logarithms
Enterprise size	Size	Fixed assets are taken as the logarithm
Enterprise age	Age	Observed year minus establishment year plus one is taken as the logarithm
Asset-liability ratio	Lev	Total liabilities/Total assets
Profitability	ROA	Net profit/Total assets
Capital expenditure	Capital	Cash paid by enterprises for purchasing fixed assets, intangible assets, and other long-term assets/Total assets
Operating cost ratio	Cost	Total operating costs/Total operating revenues
Shareholding concentration	Share	Shareholding ratio of top ten shareholders
Nature of ownership	SOE	State-owned enterprises are 1, otherwise take 0

### 4.3. Model construction

In order to verify the influence of digital transformation on heavily polluting enterprises’ GTFP, the following econometric model is set up:


(1) $$GTFP_{i,t}=\alpha_{0}+\alpha_{1}DCG_{i,t}+\alpha_{2}\mathrm{Control}s_{i,t}+\lambda_{P}+\mu_{I}+\upsilon_{Y}+\varepsilon_{i,t}$$

Where, $GTFP_{i,t}$ denotes the GTFP of enterprise i in period t , $DCG_{i,t}$ is the digital transformation of enterprise i in period t , $\mathrm{Control}s_{i,t}$ is the enterprise-level control variable, $\lambda_{P}$ , $\mu_{I}$ and $\upsilon_{Y}$ are province, industry, and year fixed effects, respectively, and $\varepsilon_{i,t}$ is the random disturbance term.

## 5. Empirical analysis

### 5.1. Descriptive statistics

Table 2 reports the descriptive statistics for the key variables in this paper. The maximum value, minimum value, mean value, and standard deviation of enterprises’ GTFP are 2.771, 0.511, 1.133, and 0.192, respectively, suggesting that there exist large gaps between various heavily polluting enterprises’ GTFP. The maximum value, minimum value, mean value, and standard deviation of enterprise digital transformation are 4.111, 0, 0.392, and 0.727, respectively, suggesting that there are also obvious differences in the digital transformation level between various heavily polluting enterprises and that some enterprises have not undertaken the digital transformation, in which their depth of digital transformation needs to be improved. The value intervals of the remaining control variables are basically the same as in previous studies.

**Table 2:** Descriptive statistics.

Variable symbol	Obs	Mean	SD	Min	Max
GTFP	3,107	1.133	0.192	0.511	2.771
DCG	3,107	0.392	0.727	0.000	4.111
Size	3,107	21.816	1.598	13.755	26.921
Age	3,107	2.822	0.325	1.099	3.738
Lev	3,107	0.479	0.187	0.059	0.852
ROA	3,107	0.043	0.052	−0.104	0.211
Capital	3,107	0.063	0.050	0.002	0.238
Cost	3,107	0.935	0.106	0.571	1.247
Share	3,107	55.519	14.976	22.260	89.650
SOE	3,107	0.678	0.467	0.000	1.000

### 5.2. Benchmark regression

Table 3 shows the effect of digital transformation on heavily polluting enterprises’ GTFP. Column (1) considers only the enterprise digital transformation (DCG) variable. As we can see, the estimation coefficient of DCG is significantly 0.0681. Column (2) includes province, industry, and year fixed effects based on column (1), and the coefficient of DCG is significant at 0.0234, and column (3) further incorporates control variables based on column (2), and the coefficient of DCG is 0.0230, which is still positive in a significant way. It indicates there is an increasing trend of heavily polluting enterprises’ GTFP as their digital transformation degree improves, and digital transformation can significantly increase such enterprises’ GTFP, which verifies Hypothesis 1 of this paper. Regarding the control variables, enterprise size (Size), capital expenditure (Capital), and operating cost rate (Cost) all have significant and negative estimated coefficients, which shows that the more fixed assets are invested, the more capital expenditure and the greater production cost, the heavier the economic burden of enterprises, the weaker the incentive for green transformation and the lower the GTFP; the estimated coefficients of enterprise age (Age), asset-liability ratio (Lev), and shareholding concentration (Share) are positive in a significant way, which means that enterprises with the longer establishment, less difficulty in raising capital, and larger shareholding of the top 10 shareholders have higher GTFP. The remaining control variables’ coefficients are not significant.

**Table 3:** Benchmark regression.

Variable	(1)	(2)	(3)
	GTFP	GTFP	GTFP
DCG	0.0681***	0.0234^***^	0.0230***
	(0.0060)	(0.0061)	(0.0058)
Size			−0.0225***
			(0.0034)
Age			0.0261*
			(0.0136)
Lev			0.0547**
			(0.0219)
ROA			0.0982
			(0.0902)
Capital			−0.2591***
			(0.0562)
Cost			−0.1576***
			(0.0514)
Share			0.0007***
			(0.0003)
SOE			0.0093
			(0.0072)
_cons	1.1060***	1.1235^***^	1.6282***
	(0.0036)	(0.0034)	(0.0904)
Province FE	NO	YES	YES
Industry FE	NO	YES	YES
Year FE	NO	YES	YES
Observations	3,107	3,107	3,107
Adj.R^2^	0.0662	0.3694	0.3903

***, **, * represent significant at the 1%, 5%, and 10% levels, respectively; robust standard errors are in parenthesis; same below.

### 5.3. Endogenous treatment

Considering that enterprises’ enhancement of GTFP is a possible motive for their digital transformation, thus the endogenous problem due to bidirectional causality may exist between the two. In this study, we utilize the instrumental variable (IV) method to alleviate the endogenous problem and test it using the 2SLS method. Drawing on Zhou et al. (2022), our instrumental variable is represented by the digital transformation degree average value of other enterprises in the same province and industry (DCG_IV). Such an instrumental variable is selected because other enterprises’ digital transformation level in the same province and industry is able to show this enterprise’s digital transformation situation, but it will not directly affect the GTFP of this enterprise, which satisfies the principles of relevance and exogeneity of the instrumental variable.

Table 4 shows the estimation results of IV method. As we can see, the LM statistic has a significant value of 169.057, which rejects insufficient identification hypothesis; and the F statistic has a significant value of 254.447, higher than the critical value corresponding to the Stock-Yogo test at the 10% level, which passes the weak identification test, indicating that our IV is appropriate. Column (1) presents the first-stage estimation results. As we can see, DCG_IV estimated coefficient is positive in a significant way, which meets the relevance requirement of instrumental variables. Column (2) presents the second-stage estimation results. As we can see, the estimated coefficient of DCG is significant at 0.0466, which means that the conclusion that enterprise digital transformation can enhance its GTFP in a significant way still holds, and the estimated result is higher than benchmark result, which shows that the endogenous problem leads to the underestimation of the enhancement role of digital transformation on enterprise GTFP, which enhances the reliability of previous conclusions and again verifies Hypothesis 1.

**Table 4:** Instrumental variable method test results.

Variable	(1)	(2)
	DCG	GTFP
DCG		0.0466***
		(0.0145)
DCG_IV	0.7103***	
	(0.0498)	
Control variables	YES	YES
FE	YES	YES
Observations	3,107	3,107
Kleibergen-Paap rk LM statistic	169.057***	
	[0.000]	
Cragg-Donald Wald F statistic	254.447***	
	{16.380}	

Same as Table 3. The value in [] is p value, and the value in {} is the critical value corresponding to the Stock-Yogo test at the 10% level.

### 5.4. Robustness test

#### 5.4.1. Replacing the explanatory variable

Replacing digital transformation measurement method and re-estimated so as to avoid the effect of measurement error. Drawing on Zhang et al. (2021), this study adopts the ratio of digitalization-related intangible assets to total enterprise intangible assets (DIG) to replace the benchmark regression’s explanatory variable and substitutes it into the model for re-estimation, which is presented in column (1) of Table 5. As we can see, after replacing the core explanatory variables, the coefficient of DIG is significant at 0.0900, which indicates that digital transformation can significantly enhance enterprise GTFP, confirming that the previous findings are robust.

**Table 5:** Robustness test.

Variable	(1)	(2)	(3)	(4)	(5)
	Replacing the explanatory variable	Replacing the explained variable	Explanatory variable lagged treatment	Replacing the estimation model	Adjusting the estimation sample
DIG	0.0900**				
	(0.0385)				
DCG		0.0216***		0.0094**	0.0609***
		(0.0059)		(0.0036)	(0.0112)
L.DCG			0.0199***		
			(0.0065)		
_cons	1.5896***	1.7956***	1.6990***	0.8219***	1.6939***
	(0.0915)	(0.0896)	(0.1020)	(0.1232)	(0.2347)
Control variables	YES	YES	YES	YES	YES
FE	YES	YES	YES	YES	YES
Observations	3,107	3,107	2,868	3,107	885
Adj.R^2^	0.3860	0.4169	0.3780		0.3867

#### 5.4.2. Replacing the explained variable

Further measuring enterprise GTFP with a depreciation rate of 9.6%, the explained variable of the benchmark regression is replaced and substituted into the model for re-estimation, which is presented in column (2) of Table 5. As we can see, the coefficient of DCG is significant at 0.0216, implying that the research finding that digital transformation is able to increase enterprise GTFP in a significant way is robust.

#### 5.4.3. Explanatory variable lagged treatment

Since the influence effect of digital transformation may be lagged, this study lags the explanatory variables by one period. Column (3) of Table 5 presents the estimation results. As we can see, the coefficient of the digital transformation indicator with one period lag (L.DCG) is significantly 0.0199, which means that GTFP in the future one period is positively related to enterprise digital transformation, the same as the conclusion above.

#### 5.4.4. Replacing the estimation model

Since the GTFP of enterprises is characterized by a left truncation at 0, the Tobit regression method with high fitness to truncated data is adopted to test. Column (4) of Table 5 presents the estimation results. As we can see, the coefficient of DCG is significant at 0.0094, which means that the previous conclusion still holds.

#### 5.4.5. Adjusting the estimation sample

Considering that several enterprises have not undergone digital transformation, which may interfere with the regression results, we exclude the sample of enterprises that have not undergone digital transformation and re-examined. Column (5) of Table 5 reports the estimation results after adjusting the estimation sample. As we can see, the conclusion that digital transformation can improve enterprise GTFP remains unchanged after excluding the sample with 0 explanatory variables, corroborating previous findings are robust.

### 5.5. Mechanism test

Based on the above discussion, digital transformation may increase heavily polluting enterprises’ GTFP by promoting green innovation, improving management efficiency, and reducing external transaction costs. To this end, the above mechanism of action is further empirically tested, and the mechanism test model is set as follows:


(2) $$\mathrm{Mechanis}m_{i,t}=\alpha_{0}+\alpha_{1}DCG_{i,t}+\alpha_{2}\mathrm{Control}s_{i,t}+\lambda_{P}+\mu_{I}+\upsilon_{Y}+\varepsilon_{i,t}$$

Where, $\mathrm{Mechanis}m_{i,t}$ denotes the mechanism variable, which contains indicators concerning green innovation, management efficiency, and external transaction costs, and other variables are consistent with model (1).

#### 5.5.1. Green innovation mechanism

We discuss the mechanism role of green innovation at two levels: green innovation quantity (GIS) and green innovation quality (GIZ), respectively. Drawing on Xiao and Zeng (2023), GIS adopts the logarithm measurement after adding one to the number of green invention patents and green utility model patents, while GIZ adopts the logarithm measurement after adding one to the number of green invention patents with high technical content. Table 6 Columns (1) and (2) examine the effects of enterprise digital transformation on green innovation quantity and green innovation quality, respectively. As we can see, the coefficients of DCG are significant at 0.0569 and 0.0539, respectively, which means that digital transformation is able to increase enterprises’ green innovation quantity and raise their green innovation quality in a significant way, that is, digital transformation is able to enhance heavily polluting enterprises’ green innovation level in a significant way. This may be because digital transformation is able to boost heavily polluting enterprises to organize green innovation resources effectively and optimize green innovation mode so as to increase their green innovation degree. According to green innovation, such enterprises can help them achieve energy conservation and pollution reduction, cost decrease, and efficiency enhancement by using clean production technology, waste treatment, and energy saving technology transformation, and then improve GTFP. Thus, there is a mechanism for digital transformation to improve heavily polluting enterprises’ GTFP by promoting their green innovation, which verifies Hypothesis 2 of this paper.

**Table 6:** Mechanism test results.

Variable	(1)	(2)	(3)	(4)
	GIS	GIZ	Manage	Asset
DCG	0.0569**	0.0539**	−0.0038***	−0.0177***
	(0.0265)	(0.0229)	(0.0010)	(0.0031)
_cons	−4.1967***	−3.4651***	0.1893***	−0.2637***
	(0.4337)	(0.3661)	(0.0213)	(0.0689)
Control variables	YES	YES	YES	YES
FE	YES	YES	YES	YES
Observations	3,107	3,107	3,107	3,107
Adj.*R*^2^	0.4036	0.3201	0.4772	0.5726

#### 5.5.2. Management efficiency mechanism

Management efficiency (Manage) is measured using the management expense ratio, where a lower management expense ratio indicates a higher management efficiency. Column (3) of Table 6 reports the findings of enterprise digital transformation and management expense ratio. As we can see, the estimated coefficient of DCG is significant at −0.0038, which means that digital transformation is able to reduce heavily polluting companies’ management expense ratio significantly, that is to say, digital transformation is able to increase heavily polluting companies’ management efficiency in a significant way. This is because heavily polluting companies deeply integrate data resources, digital platforms, and digital technologies with their management fields through digital transformation, which optimizes their operation and management modes, enhances the transparency and visualization of their various management processes, reduces their management costs, and improves their management efficiency. The improvement of management efficiency helps to increase heavily polluting enterprises’ resource utilization efficiency and technological innovation degree, which in turn improves GTFP. Therefore, there is a mechanism for digital transformation to improve heavily polluting enterprises’ GTFP by improving their management efficiency, which verifies Hypothesis 3 of this paper.

#### 5.5.3. External transaction cost mechanism

External transaction costs (Asset) adopt asset specificity as a proxy indicator, specifically adopting the proportion of fixed assets to total assets to define, and the higher the asset specificity shows that enterprises face higher external transaction costs. Column (4) of Table 6 reports the related estimation results. As we can see, the coefficient of DCG is significant at −0.0177, which shows that digital transformation is able to significantly reduce heavily polluting enterprises’ asset specificity level, in other words, digital transformation could significantly decrease the external transaction costs faced by heavily polluting companies. The reason may be that digital transformation could effectively decrease heavily polluting enterprises’ internal and external information asymmetry and reduce their external transaction costs such as information search cost, negotiation cost, supervision cost, and default cost of enterprises. The reduction of external transaction costs can help improve the operational efficiency and profitability of heavily polluting enterprises, relieve heavily polluting enterprises’ financing constraints, stimulate their green transformation motivation, and ultimately improve GTFP. Thus, there is a mechanism for digital transformation to improve heavily polluting enterprises’ GTFP by reducing their external transaction cost, which verifies Hypothesis 4 of this paper.

### 5.6. Heterogeneity analysis

The previous paper verified that digital transformation could enhance heavily polluting enterprises’ GTFP in a significant way. Next, the heterogeneity characteristics exhibited by its impact are further discussed based on enterprise property rights nature, industry science and technology attributes, geographic regions, and structural characteristics of digital transformation.

#### 5.6.1. Heterogeneity of enterprise property rights nature

The study sample is classified into state-owned and non-state-owned enterprises according to enterprise property rights nature. Columns (1) and (2) of Table 7 demonstrate the relevant estimation results. As we can see, the coefficients of DCG are all positive at the 1% level in a significant way, which means that digital transformation is able to significantly enhance heavily polluting enterprises’ GTFP in both types of enterprises, but its enhancement effect is greater in non-state-owned enterprises. This is because state-owned enterprises own more financial and policy advantages, which makes them face less competitive pressure and thus lack the incentive to innovate, and their management mode and operation mechanism are more solidified, so their digital transformation process is slow. However, non-state-owned enterprises are self-financing, which have more incentive to implement digital transformation under the pressure of fierce competition, thus better improving their GTFP.

**Table 7:** Heterogeneity analysis results based on enterprise property rights nature, industry science and technology attributes, and geographic regions.

Variable	(1)	(2)	(3)	(4)	(5)	(6)
	State-owned enterprises	Non-state-owned enterprises	High-tech industries	Non-high-tech industries	Eastern region	Central and western regions
DCG	0.0196**	0.0297***	0.0156**	0.0307***	0.0344***	0.0088
	(0.0079)	(0.0074)	(0.0064)	(0.0116)	(0.0070)	(0.0093)
_cons	1.7120***	1.2172***	1.2091***	2.4159***	1.3609***	1.7606***
	(0.1146)	(0.1545)	(0.1088)	(0.1751)	(0.1223)	(0.1470)
Control variables	YES	YES	YES	YES	YES	YES
FE	YES	YES	YES	YES	YES	YES
Observations	2,107	1,000	1840	1,267	1,617	1,490
Adj.*R*^2^	0.4096	0.5042	0.3787	0.4647	0.4008	0.4084

#### 5.6.2. Heterogeneity of industry science and technology attributes

Drawing on the study by Peng and Mao (2017), the study sample is divided into high-tech and non-high-tech industries. Columns (3) and (4) of Table 7 present the group estimation results. As we can see, the coefficients of DCG are all positive at the 1% level in a significant way, which means digital transformation has significantly positive effects on heavily polluting enterprises’ GTFP in both types of industries, but its improvement is more obvious in non-high-tech industries. This may be due to the fact that technology innovation activities are more frequent in high-tech industries, in which heavily polluting enterprises have technology advantages, their digital development level is usually higher, and the space for digital transformation to play a further role is limited, thus the effect of improving GTFP through digital transformation is weaker. In contrast, heavily polluting companies in non-high-tech industries often have a much lower level of digital development and more room for digital transformation to play, thus showing a more obvious improvement role of digital transformation on their GTFP.

#### 5.6.3. Heterogeneity of geographic regions

The study sample is classified into the eastern region and the central and western regions based on the location of heavily polluting enterprises. Columns (5) and (6) of Table 7 report the relevant grouping results. As we can see, the coefficient of DCG is positive in a significant way only in the eastern region, while it is not significant in the central and western regions, which means there is a more obvious enhancement role of digital transformation on heavily polluting enterprises’ GTFP in the eastern region. This may be due to the fact that the eastern region holds relative benefits in terms of economic development and digital infrastructure construction, and heavily polluting enterprises in the eastern region are able to get more digital development opportunities, and face more fierce market competition, usually have stronger awareness of technology innovation, and have more motivation to implement digital transformation, reduce external transaction costs and improve internal governance structure, and thus improve GTFP more significantly. However, the economic development degree of the central and western regions is lower, and the digital infrastructure construction is not perfect enough, so heavily polluting enterprises’ digital transformation space in the central and western regions is comparatively limited, which results in the limited role of their digital transformation in enhancing GTFP.

#### 5.6.4. Heterogeneity of structural characteristics of digital transformation

Particularly, considering that digital transformation contains technological differences with different structural characteristics (Wu et al., 2021), it may differentially affect enterprise GTFP. Therefore, we examine the role of the five segmentation indicators of digital transformation on heavily polluting enterprises’ GTFP, respectively. Table 8 presents the related estimation results. As we can find, the coefficients of AI, CC, DT, and ADT are all positive in a significant way, while the coefficient of BD is positive but not significant, which suggests that AI, CC, DT, and ADT are more significant than BD in contributing to the heavily polluting enterprises’ GTFP improvement. The possible reason is that the mining and utilization of data by frontier information technology are helpful for optimizing heavily polluting enterprises’ business processes and management modes, therefore, strengthening to develop and apply frontier information technology could encourage such enterprises to undertake technological innovations, reduce production costs and transaction costs, improve operational efficiency, and thus improve their GTFP. Among them, the improvement effect of BD on enterprise GTFP is not significant, probably because the current domestic BD is not mature, and it has not achieved a breakthrough in both scale and feasibility, and some technology applications suffer from policy barriers, which makes it difficult to be valued by enterprises, so it has no significant improvement role in enterprise GTFP.

**Table 8:** Heterogeneity analysis results based on structural characteristics of digital transformation.

Variable	(1)	(2)	(3)	(4)	(5)
	GTFP	GTFP	GTFP	GTFP	GTFP
AI	0.0426*				
	(0.0248)				
BD		0.0710			
		(0.0734)			
CC			0.0613***		
			(0.0183)		
DT				0.0361***	
				(0.0114)	
ADT					0.0198***
					(0.0062)
_cons	1.6106***	1.6060***	1.6062***	1.6191***	1.6253***
	(0.0916)	(0.0916)	(0.0909)	(0.0911)	(0.0902)
Control variables	YES	YES	YES	YES	YES
FE	YES	YES	YES	YES	YES
Observations	3,107	3,107	3,107	3,107	3,107
Adj.*R*^2^	0.3861	0.3853	0.3913	0.3888	0.3880

## 6. Conclusions and policy suggestions

Digital transformation is a crucial engine for enhancing heavily polluting enterprises’ GTFP. According to the theoretical analysis, we use Chinese A-share listed enterprises in the heavily polluting industry data from 2007 to 2019, measure enterprise digital transformation indicator using text analysis, and measure enterprise GTFP indicator using the GML index on the basis of the SBM directional distance function, so as to verify the effect of digital transformation on heavily polluting enterprises’ GTFP and its mechanism. Our research conclusions mainly include: First, digital transformation is able to significantly enhance heavily polluting enterprises’ GTFP, and the finding remains valid after considering the endogenous problem and conducting the robustness tests. Second, digital transformation is able to enhance heavily polluting enterprises’ GTFP by promoting green innovation, improving management efficiency, and reducing external transaction costs. Third, the role of digital transformation in enhancing heavily polluting enterprises’ GTFP has obvious differences according to enterprise property rights nature, industry science and technology attributes, geographic regions, and structural characteristics of digital transformation. The improvement role of digital transformation on heavily polluting enterprises’ GTFP is more obvious in non-state-owned enterprises and non-high-tech industries; the improvement role of digital transformation on heavily polluting enterprises’ GTFP is significant in the eastern region but not significant in the central and western regions; artificial intelligence technology, cloud computing technology, big data technology, and digital technology application can significantly improve heavily polluting enterprises’ GTFP, while blockchain technology has no significant improvement role.

Corresponding to the above research conclusions, three policy suggestions are obtained:

First, accelerate heavily polluting enterprises’ digital transformation, and add new power to enhance GTFP with digital transformation. Research has found that digital transformation can bring green and efficiency changes to heavily polluting enterprises, helping to improve their GTFP. Therefore, the government should increase policy support for heavily polluting enterprises’ digital transformation, and through tax incentives, financial subsidies, and talent introduction to help heavily polluting enterprises complete their digitalization and intelligent transformation, so as to better release digital transformation dividends. Meanwhile, accelerate to improve digital infrastructure construction, strengthen high-quality data elements supply, promote the penetration of digital technology to heavily polluting enterprises, and consolidate the digital foundation of their green transformation. In addition, heavily polluting enterprises should actively invest in digital construction, systematically plan digital transformation strategies suitable for their own green development, and increase investment in digital transformation funds and talents, deeply integrate digital technology with their own production, R&D, management, and other links to enhance the digital transformation degree of the core links, and give full play to the role of digital transformation to empower green development.

Second, smooth the transmission path of digital transformation and promote heavily polluting enterprises’ green transformation. According to the research results, the key mechanisms of digital transformation to enhance heavily polluting enterprises’ GTFP lie in promoting green innovation, improving management efficiency, and reducing external transaction costs. Therefore, heavily polluting enterprises should focus on empowering green innovation with digital technology, strengthening the application of digital technology in the green innovation process of clean production, end treatment, and energy saving type, enhancing green innovation capability, promoting energy conservation, pollution reduction, cost reduction, and efficiency improvement, so as to realize green transformation. Meanwhile, digital technology should be used to innovate traditional organizational structure and management modes, integrate digital technology into operation and management fields, establish efficient cross-department and cross-organizational collaboration mechanisms, realize information sharing and collaboration, and increase digital management levels, and give full play to digital empowerment in green transformation process. In addition, the institutional environment is the decisive factor in external transaction costs. The government should strengthen administrative system reform, improve governance efficiency, promote the “effective market” with “competent government,” continuously improve the business environment and contract environment, so as to defuse heavily polluting enterprises’ external transaction risks, reduce their external transaction costs, and create conducive conditions for their green transformation.

Third, precisely applying policies to help various types of heavily polluting enterprises implement digital transformation in a smooth and orderly manner. It is found that the influence of digital transformation on heavily polluting enterprises’ GTFP varies among enterprise characteristics. Therefore, the government should follow differentiation principles and implement accurate digital transformation support policies according to enterprise property rights nature, industry science and technology attributes, as well as geographic regions characteristics. Specifically, for state-owned heavily polluting enterprises, they should be guided to use digital technology to promote deeper changes in management mode and operation mechanism, and take digital transformation as a major method to achieve high-quality development. For heavily polluting enterprises in high-tech industries, encourage them to grasp digital economy advantages and explore digital transformation solutions suitable for their green development. Meanwhile, increase the construction of digital infrastructure in the central and western regions, accelerate “East Digital West Computing” project, and increase the overall level of national computing power so as to better empower digital development. In particular, according to the research findings, since blockchain technology has not yet achieved a technological breakthrough in both scale and feasibility, and cannot significantly improve GTFP, the government should improve the laws and regulations in blockchain technology field, increase the research and development of blockchain underlying core technology, and promote heavily polluting enterprises to efficiently apply blockchain technology to upgrade their green development.

In addition, this paper has limitations that can be further explored in future research. First, this paper focuses on the sample of listed companies in Chinese heavily polluting industries, which cannot well reflect the situation in other countries and other industries, future research can expand to analyze other countries and other industries. Second, this paper uses the way of analyzing the textual information related to enterprise annual reports to measure digital transformation, the results may have a gap with the actual, future research can construct multi-dimensional indicators to measure. Third, the mechanism is only explored from the dimensions of green innovation, management efficiency, and external transaction costs, but the influence of digital transformation is extensive, future research can explore other influence paths.

## Data availability statement

The raw data supporting the conclusions of this article will be made available by the authors, without undue reservation.

## Author contributions

JH: Data curation, Project administration, Writing – original draft, Writing – review & editing. RS: Writing – review & editing, Conceptualization, Methodology, Validation. MZ: Conceptualization, Investigation, Software, Writing – review & editing. AR: Data curation, Formal analysis, Software, Writing – review & editing. IU: Project administration, Visualization, Conceptualization, Writing – original draft.
